# Cumulative effect of multiple health and social factors on adolescent mental well‐being: a cross‐sectional study in Catalonia

**DOI:** 10.1111/camh.70070

**Published:** 2026-01-14

**Authors:** Marina Robles‐Muñoz, Marina Bosque‐Prous, Cinta Folch, Helena González‐Casals, Gemma Drou‐Roget, Cristina Torrado‐Cortés, Carles Muntaner, Albert Espelt

**Affiliations:** ^1^ Epi4Health, Department of Psychobiology and Methodology in Health Sciences Universitat Autònoma de Barcelona (UAB) Bellaterra Spain; ^2^ Sant Andreu Salut Manresa Spain; ^3^ Centro de Investigación Biomédica en Red de Epidemiología y Salud Pública (CIBERESP) Madrid Spain; ^4^ Centre d'Estudis Epidemiològics sobre les Infeccions de Transmissió Sexual i Sida de Catalunya (CEEISCAT), Departament de Salut. Generalitat de Catalunya Badalona Spain; ^5^ Institut d'Investigació Germans Trias i Pujol (IGTP) Badalona Spain; ^6^ Departament de Salut Generalitat de Catalunya Barcelona Spain; ^7^ Epi4health, Faculty of Health Sciences Universitat Oberta de Catalunya Barcelona Spain; ^8^ Department of Basic, Developmental and Educational Psychology Universitat Auntònoma de Barcelona Bellaterra Spain; ^9^ BFON, University of Toronto Toronto ON Canada; ^10^ Hopkins – Universitat Pompeu Fabra (JHU‐UPF) Public Policy Center and Barcelona School of Management (BSM), Universitat Pompeu Fabra Barcelona Spain

**Keywords:** Adolescence, mental health, risk factors, public health

## Abstract

**Background:**

While numerous studies have explored factors contributing to poor mental health, few have examined the combined influence of multiple health and social factors and their cumulative effect. This study specifically aimed to analyze the cumulative effect of multiple health and social factors associated with poor mental well‐being in school‐aged adolescents (12–18 years old) in Central Catalonia during the 2021–2022 academic year.

**Methods:**

Cross‐sectional study using data from the 2021–2022 DESKcohort project, with a total sample of 9265 high‐school aged students in Central Catalonia (49.2% boys and 50.8% girls). To identify factors associated with poor mental well‐being (score < 44 on the Warwick‐Edinburgh Mental Well‐being Scale), Multilevel Poisson regression models with robust variance were estimated, obtaining prevalence ratios (PR) with their respective 95% confidence intervals (95% CI). The cumulative effect score was derived by summing the factors that were associated with poor mental well‐being.

**Results:**

We observe a gradient where the likelihood of experiencing poor mental well‐being increases statistically significantly with exposure to a greater number of multiple health and social factors. In boys, the prevalence ratio increases statistically significantly from PR = 1.59 (*p* < .040) to PR = 24.52 (*p* < .001) for 1 and 10 health and social factors combined, and in girls, increases statistically significantly from PR = 2.56 (*p* < .001) to PR = 16.23 (*p* < .001) for 1 and 11 health and social factors combined, respectively.

**Conclusion:**

The findings of this study highlight the multifaceted nature of mental health and the co‐presence of multiple axes of inequality, where overall health, family and psychosocial factors, and health behaviors form a cumulative association with adolescents' poor mental well‐being.

**Key Practitioner Message**

**What is currently known?**

- Mental health is a major public health problem worldwide, and adolescence is a critical period which can increase the vulnerability to develop mental health problems.

**What has been shown?**

- Numerous studies have examined individual factors linked to poor adolescent mental well‐being, but few have assessed their cumulative impact using large, population‐based samples including both rural and urban areas. In the absence of a systematic review, we acknowledge such studies may exist but seem scarce.
- Using data from the DESKcohort project, we aimed to contribute to the better understanding of adolescent mental health by describing the cumulative effect drawn from the presence of multiple determinants of mental health on adolescent mental well‐being, rather than targeting their effect in isolation.

**What is the significance of this for clinical practice?**

- We found the existence of a dose–response gradient indicating that the prevalence of poor mental well‐being increases as adolescents are exposed to a greater number of health and social factors, including multiple forms of inequality, overall health, psychosocial factors, and health behaviors.
- These findings encourage holistic and preventive interventions that prioritize modifiable health and social risk factors rather than more complex, immediate causes of distress, which can create positive cascade effects in mental health outcomes.

## Introduction

Poor mental health in adolescence is one of the major public health problems worldwide. Adolescence is a critical period marked by substantial physical, emotional, and social changes, which can increase the vulnerability to develop mental health problems (WHO, 2024). In Spain, in 2021, 17.7% of adolescents aged 15–19 frequently experienced mental health issues (Sanmartín, Ballesteros, Calderón, & Kuric, 2022). In turn, poor mental health in youth can lead to a range of negative outcomes, including poor physical health, social exclusion, discrimination, stigma, educational difficulties, and the adoption of risk‐taking behaviors (WHO, 2024).

A significant number of studies have identified a long list of biological, psychological, and social determinants linked to poor mental health. For instance, as adolescents transition into adulthood, their health‐related behaviors (e.g., adequate sleep and exercise habits, eating breakfast, maintaining a healthy weight, the use of addictive substances) become less healthy (Frech, 2012; Rogés et al., 2023). Not only are these behavioral patterns associated with poorer mental health (Codinach‐Danés et al., 2024; Díaz‐Geada et al., 2020; Hutchesson et al., 2023; Mangot‐Sala et al., 2019; Wickham, Amarasekara, Bartonicek, & Conner, 2020), but they can also persist into adulthood (Sawyer et al., 2012).

A recent conceptual framework for public mental health consolidated 55 determinants linked to mental health across individual, family, community, and structural levels (Dykxhoorn et al., 2022). Building upon this multifaceted nature of mental health, recent studies have highlighted the importance of considering the cumulative effect of mental health determinants rather than analyzing them in isolation and have shown that when adolescents are exposed to multiple risk factors, their likelihood of experiencing poor mental health significantly increases compared to those exposed to just one factor (Appleyard, Egeland, Van Dulmen, & Alan Sroufe, 2005; Deater‐Deckard, Dodge, Bates, & Pettit, 1998; Demkowicz, Panayiotou, & Humphrey, 2021; Raviv, Taussig, Culhane, & Garrido, 2010; Wang, Lu, Zhao, Li, & Zhou, 2024).

Within this framework, the syndemic theory emerges as a particularly relevant model for examining adolescent mental well‐being. This theory reflects the idea that multiple health‐related determinants do not merely coexist, but also interact with each other, exacerbating their individual impacts on the health status of individuals and populations (Singer & Clair, 2003). Despite the growing recognition of syndemics in literature, there is still limited research focusing on adolescents, particularly in the context of poor mental well‐being. Although the term syndemic implies the existence of interactions between factors, many studies, including this one, focus on the cumulative effect of multiple risk factors, which can be seen as a step toward identifying syndemic patterns (Singer, Bulled, & Ostrach, 2020).

The relevance of this study lies in the exploration of the cumulative effect of a wide range of mental health determinants – including overall health, axes of inequality (such as sexual orientation and migratory status), psychosocial factors (such as the quality of family relationships and experiences of bullying and sexual violence), and health‐related behaviors – on a large sample of adolescents from both rural and urban areas. Central Catalonia was selected due to its diverse territorial profile, with a variety of social and geographical contexts, in contrast to most studies focused on large metropolitan areas. For instance, some studies have shown that alcohol‐related risk behaviors differ between rural and urban environments (Font‐Ribera et al., 2013). Detecting cumulative factors can be useful for the development of holistic and preventive interventions that address the health challenges that appear at this stage, especially over those determinants that are more easily amenable to change through public health actions (Dykxhoorn et al., 2022). We hypothesize that greater cumulative exposure to social and health‐related factors is associated with poorer mental well‐being among adolescents. Thus, our study aimed to analyze the cumulative effect of multiple health and social factors associated with poor mental well‐being in school‐aged adolescents (12–18 years old) in Central Catalonia during the 2021–2022 academic year.

## Methods

We conducted a cross‐sectional study using the data from the second wave (2021–2022) of the DESKcohort Project, a prospective longitudinal study with successive waves that allows describing and monitoring health, health behaviors, and their related factors in school‐aged adolescents in Central Catalonia (Rogés et al., 2023). We adhered to the STROBE reporting guidelines (see Appendix S1).

### Participants

The sample was a convenience sample consisting of 12‐ to 18‐year‐old students enrolled in high schools in Central Catalonia, attending the following grade levels: second and fourth of Compulsory Secondary Education (CSE); second of Post‐Compulsory Secondary Education (PCSE); and second of Intermediate Level Training Cycles (ILTC). The final sample consisted of 9265 students aged 12–18 years, of whom 49.2% were boys and 50.8% were girls.

### Data collection tools and procedures

The DESKcohort survey was created to study adolescent health in a holistic manner, with the collaboration of professionals from different health fields. It was based on similar surveys with high validity and reliability, and it includes validated scales and other questions developed by a multidisciplinary panel of experts. It is an ad hoc self‐administered questionnaire that was answered in person and in classrooms using tablets. Data from the participants were collected between October 2021 and June 2022. All educational centers (*n* = 98) were invited to participate through a letter, and those that agreed to participate (*n* = 84) were sent informed consents to be signed by all participants and the participants' legal guardians for those under 14 years old. A total of 9265 out of 14,324 adolescents participated in the study (64.6%) (Rogés et al., 2023).

### Ethical aspects

The project was approved by the Ethics Committee of the Universitat de Vic‐Universitat Central de Catalunya (UVic‐UCC) (96/2019). For questions that could be sensitive or problematic (especially those involving possible traumatization), the team responsible for administering the survey was trained and prepared with resources to respond in case participants experienced discomfort or had any concerns. No incidents requiring intervention were reported. They referred participants to the person in charge of the school's psycho‐pedagogical department for appropriate follow‐up and offered resources from the Health Department of the Government of Catalonia. This was made possible because the study involved a working group that included the Public Health Agencies of Catalonia and Barcelona, the Departments of Education of Catalonia and Central Catalonia, and organizations experienced in dealing with risk behaviors, such as the Center for Epidemiological Studies on HIV/AIDS in Catalonia (CEEISCAT), Agència de Salut Pública de Barcelona, Universitat de Vic‐Universitat Central de Catalunya, and Universitat Autònoma de Barcelona.

### Measures

#### Outcome variables

The outcome variable of the study was *poor mental well‐being*, measured with the Spanish adaptation of the Warwick‐Edinburgh Mental Well‐being 14‐item scale (WEMWBS) (López et al., 2013). We used the cutoff score of 44, as proposed by the authors of the scale, who suggest that scores below 44 indicate possible or mild depression (https://warwick.ac.uk/services/innovations/wemwbs/). In our sample, the reliability of the WEMWBS, as measured by Cronbach's alpha coefficient (α), was .89 (95% CI: 0.89–0.90). To assess validity, a Structural equation model with mental well‐being as a latent variable with Satorra‐Bentler estimation was fitted obtaining good estimations (RSMEA 0.08, CFI 0.90, TLI 0.88, and SRMR 0.05).

#### Exposure variables

**Figure 1:**
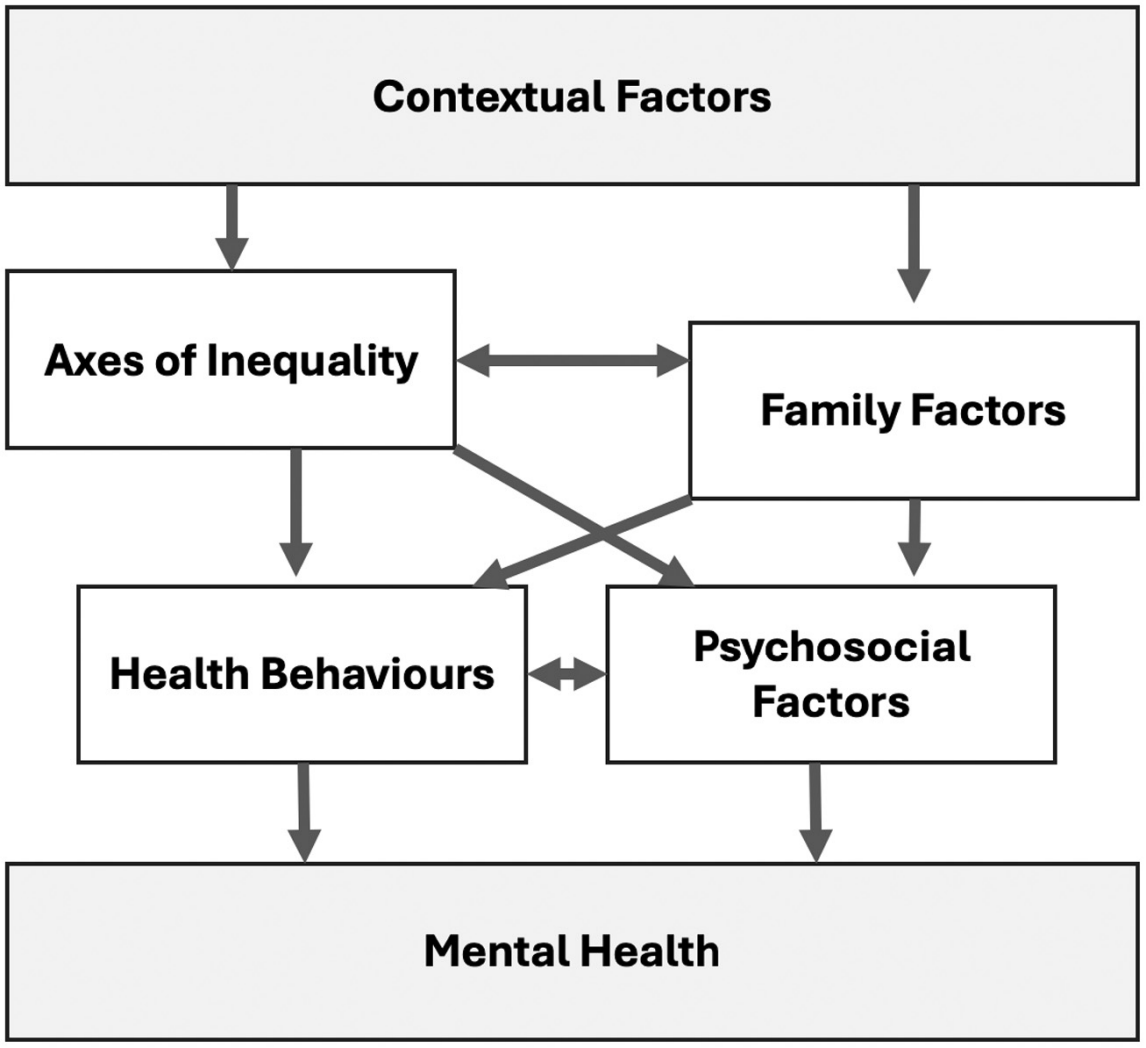
Framework of the determinants of mental health in adolescents. Adapted from the conceptual framework for public mental health and the conceptual framework on the determinants of social inequalities (Comisión Para Reducir Las Desigualdades Sociales En Salud En España, 2012; Dykxhoorn et al., 2022)

The selection of exposure variables was guided by the public mental health conceptual framework (Dykxhoorn et al., 2022) and determined by the available items in the DESKcohort survey. Inspired by the framework on the social determinants of health inequities (Comisión Para Reducir Las Desigualdades Sociales En Salud En España, 2012), these variables are grouped in categories illustrated in Figure 1, including contextual factors, axes of inequality, psychosocial factors, and health behaviors.

- Contextual factors

*Size of municipality* as a proxy for contextual determinants of health. Territories with fewer than 10,000 inhabitants are classified as rural, while those with more than 10,000 inhabitants, as urban.

ii Axes of inequality

Axes of inequality included *gender* (girl/boy), *grade level* as an indirect measure for age (“2^nd^ and 4^th^ courses of CSE” and “2^nd^ course of PCSE and ILTC”), *subjective socioeconomic position or SEP* (“advantaged or average” and “deprived”), measured using the MacArthur Scale of Subjective Social Status where higher values indicated more advantaged SEP (Goodman et al., 2001), *migratory status* (“native” and “1^st^ or 2^nd^ generation immigrant”), *sexual orientation* (“heteronormative” and “nonheteronormative”), and *self‐reported academic performance* (“good or average grades” and “poor grades”).

iii Psychosocial factors

Psychosocial factors included *having experienced bullying* (yes/no), *sexual violence* (no/yes or prefer not to answer), and *quality of family relationships* (“very good or good” and “fair, poor or very poor”). Bullying victims were defined by the frequency of experiences such as being laughed at/insulted, excluded, or being physically attacked or threatened at school or on their way to school in the past year. There were five possible responses (“never” to “4 or more times”). Victims were considered if they had experienced all situations at least once during the past year, or a total of 4 or more instances of any of the situations. Sexual violence victims were considered if they reported experiencing any of the following situations during their lifetime: “Without physical contact (sexist jokes with sexual content, unwanted continuous sexualized looks, exhibitionism, etc.),” “With physical contact (continuous invasion of personal space, unwanted touching, cornering with sexual intentions, etc.),” and “With the introduction of objects or any part of their body into your body, whether orally, anally, or vaginally (rape with or without force, etc.).”

iv Health status

*Self‐perceived overall health* was measured with the question: “How would you rate your health in general?” and responses were divided into the following categories: “excellent, very good or good” and “fair or poor.”

v Health behaviors

Finally, health behaviors included *physical activity, sleep quality, diet quality, hazardous alcohol use*, *hazardous cannabis use, and problematic internet use. Physical activity* was measured by whether the World Health Organization (WHO) recommendations of >60 min/day were met or not (WHO, 2010); *sleep quality* was created from the question: “During the last month, how would you rate, in general, the quality of your sleep?” (“very good or good” or “poor or very poor”); *diet quality* was created by collecting information on food consumption using a food frequency questionnaire and estimating the quality of diet using the Spanish adaptation of the Healthy Eating Index (Norte‐Navarro & Ortiz‐Moncada, 2011). The score was then categorized into two groups based on their scores: “unhealthy diet or needs changes” (<80 points) or “healthy diet” (>80 points). Substance‐use behaviors included *hazardous alcohol use*, with a score above 3 on the Alcohol Use Disorders Identification Test (AUDIT‐C, Cronbach's α = .83) (Liskola et al., 2018), and *hazardous cannabis use*, with a score above 7 on the Cannabis Abuse Screening Test (CAST‐F, Cronbach's α = .89) (Cuenca‐Royo et al., 2012). Finally, *problematic internet use* was considered with a score above 28 on the Compulsive Internet Use Scale (CIUS, Cronbach's α = .88) (Ortuño‐Sierra, Pérez‐Sáenz, Mason, Pérez De Albeniz, & Fonseca Pedrero, 2024).

### Data analysis

First, we examined the distribution of sample characteristics by gender. Next, we determined the prevalence of poor mental well‐being for each exposure variable to understand the potential connections between poor mental well‐being and its underlying factors. Then, we used crude and adjusted Multilevel Poisson regression models with robust variance (i.e., robust standard errors) and school random effects to identify which factors were associated with poor mental well‐being, obtaining prevalence ratios (PR) with their respective 95% CI (Espelt, Mari‐Dell'Olmo, Penelo, & Bosque‐Prous, 2017). Crude models refer to bivariate analyses assessing associations between each exposure and the outcome without adjusting for other variables, whereas adjusted models refer to multivariable analyses that include all exposure variables simultaneously. To determine the final models, all statistically significant variables from the crude regression analyses were included in the adjusted models. Model selection was performed using a backward stepwise approach, with the 95% confidence interval and *p*‐value serving as indicators of significance. Ultimately, only variables with a *p*‐value below .05 were retained in the final model. Finally, a cumulative effect score was calculated as the sum of the 12 factors that were significantly associated with poor mental well‐being in both genders. Each exposure variable was dichotomized (1 = higher risk, 0 = lower or no risk), and a cumulative score was computed by summing the number of high‐risk exposures for each participant. This resulted in a total score ranging from 0 (no risk factors present) to 12 (maximum number of risk factors considered). This can be expressed as

$$\text{Cumulative score}=\sum_{i}\;x_{i}$$

where *xᵢ* = 1 if the individual is exposed to risk factor *i*, and *xᵢ* = 0 otherwise. The cumulative score was then included as an independent variable in Poisson regression models to assess the dose–response relationship with poor mental well‐being.

All analyses were disaggregated by gender, in line with the approach promoted by the Public Health Agency of Barcelona to ensure a gender‐sensitive interpretation of the results (Borrell & Artazcoz, 2008). Missing data were observed in only three variables – academic performance, migratory status, and problematic internet use – each with less than 5% missing values. Given the low proportion of missingness, we proceeded with the analysis by excluding cases with incomplete data. Analyses were conducted using STATA18 software (do‐file available at 10.5281/zenodo.15806983).

## Results

The total sample included 9265 students, with 49.2% boys and 50.8% girls. Among the total sample of students who reported experiencing poor mental well‐being (29.9%), a statistically significant higher prevalence was observed among girls compared to boys (41.6% vs. 17.8%, respectively, *p* < .001). Additionally, statistically significant gender differences were observed across most exposure variables, except for size of municipality (*p* = .952), subjective SEP (*p* = .602), migratory status (*p* = .491), diet quality (*p* = .313), and hazardous cannabis use (*p* = .744) (Table 1).

**Table 1:** Characteristics of school‐aged adolescents in Central Catalonia in the 2021–2022 academic year, according to gender

		Boys, *N* (%)		Girls, *N* (%)		
	Total	4558	49.2	4707	50.8	
	Poor mental well‐being					
	No	3745	82.2	2751	58.4	**<.001**
	Yes	813	17.8	1956	41.6	
Overall health	Self‐perceived overall health					
	Excellent, very good, or good	4290	94.1	4007	85.1	**<.001**
	Fair or poor	268	5.9	700	14.9	
Contextual factor	Size of municipality					
	Rural	2352	52.7	2452	52.8	.95
	Urban	2112	47.3	2172	47.2	
Axes of inequality	Grade level					
	Second and fourth course of CSE	3210	70.4	3201	68	.**01**
	Second course of PCSE and ILTC	1348	29.6	1506	32	
	Subjective SEP					
	Advantaged or Medium	2977	65.3	3050	64.8	.6
	Deprived	1581	34.7	1657	35.2	
	Migratory status					
	Native	3343	76.2	3410	75.5	.49
	First‐ or second‐generation immigrant	1046	23.8	1104	24.5	
	Sexual orientation					
	Heteronormative	3980	87.3	3154	67	**<.001**
	No heteronormative	578	12.7	1553	33	
	Academic performance					
	Good or average	3767	87.1	4115	91.5	**<.001**
	Poor	556	12.9	383	8.5	
Psychosocial factors	Bullying					
	No	3901	85.6	3804	80.8	**<.001**
	Yes	657	14.4	903	19.2	
	Sexual violence					
	No	3969	88	2033	43.7	**<.001**
	Yes/prefer not to answer	541	12	2623	56.3	
	Quality of family relationships					
	Very good or good	4044	88.7	3611	76.7	**<.001**
	Fair, poor, or very poor	514	11.3	1096	23.3	
Health behaviors	Physical activity					
	Meets WHO recommendations	2615	57.4	1752	37.2	**<.001**
	Does not meet WHO recommendations	1943	42.6	2955	62.8	
	Sleep quality					
	Very good or good	3337	73.2	2775	59	**<.001**
	Poor or very poor	1221	26.8	1932	41	
	Diet quality					
	Healthy or needs changes	4287	94.1	4450	94.5	.31
	Unhealthy	271	5.9	257	5.5	
	Hazardous alcohol use					
	No	3965	87	3816	81.1	**<.001**
	Yes	593	13	891	18.9	
	Hazardous cannabis use					
	No	4426	97.1	4576	97.2	.74
	Yes	132	2.9	131	2.8	
	Problematic internet use					
	No	3730	82.2	3368	71.7	**<.001**
	Yes	809	17.8	1331	28.3	

*p*‐values highlighted in bold indicate Prevalence Ratios that are statistically significant.

In Tables 2 and 3, statistically significant gender differences were observed across all factors, with girls consistently exhibiting higher prevalence rates of poor mental well‐being in all variables compared to boys. Students reporting a fair or poor self‐perceived overall health showed the highest prevalence of poor mental well‐being in both genders (54.1% [95% CI 48.1–60.0] in boys and 78% [95% CI 74.8–80.9] in girls), followed by those reporting fair, poor, or very poor family relationships (46.5% [95% CI 42.2–50.8] in boys and 70% [95% CI 67.2–72.6] in girls). We observed that perceived overall health, axes of inequality, psychosocial factors, and many health behaviors were statistically significantly associated with poor mental well‐being. Gender inequalities were found in the adjusted models: overall, higher aPRs were found among boys. Exclusively for boys, poor mental well‐being was also statistically significantly associated with attending second course of PCSE or ILTC (aPR = 1.18 [95% CI 1.03–1.36]), whereas for girls, results showed a unique association with being first‐ or second‐generation migrant (aPR = 1.15 [95% CI 1.03–1.28]). No statistically significant associations were found either with size of municipality and hazardous alcohol or cannabis use across both genders. Factors with the highest aPRs coincided for both genders: reporting a very poor or poor sleep quality (aPR = 2.08 [95% CI 1.85–2.35] in boys and aPR = 1.52 [95% CI 1.41–1.64] in girls), regular, poor, or very poor quality of family relationships (aPR = 1.88, [95% CI 1.66–2.10] in boys and aPR = 1.57, [95% CI 1.47–1.66] in girls), and regular, poor, or very poor self‐perceived health (aPR = 1.73, [95% CI 1.47–2.04] in boys and aPR = 1.43, [95% CI 1.32–1.55] in girls).

**Table 2:** Prevalence of poor mental well‐being and crude and adjusted prevalence ratios (PR and aPR) for each factor associated with poor mental well‐being in school‐aged boys in Central Catalonia in the 2021–2022 academic year (*N* = 4558)

		*N*	% and 95% CI	PR	95% CI	aPR	95% CI
Overall health	Self‐perceived overall health						
	Excellent or very good	4290	15.6 (14.5–16.7)	1.00		1.00	
	Regular, poor or very poor	268	54.1 (48.1–60.0)	3.47	[3.05–3.96]	1.73	[1.47–2.04]
Contextual factor	Size of municipality						
	Rural	2352	16.9 (15.5–18.5)	1.00			
	Urban	2112	18.8 (17.1–20.5)	1.11	[0.98–1.27]		
Axes of inequality	Grade level						
	Second and fourth course of CSE	3210	16.2 (15.0–17.5)	1.00		1.00	
	Second course of PCSE and ILTC	1348	21.7 (19.6–24.0)	1.35	[1.17–1.55]	1.15	[1.01–1.31]
	Subjective SEP						
	Advantaged or average	2977	14.5 (13.3–15.9)	1.00		1.00	
	Deprived	1581	24.0 (22.0–26.2)	1.65	[1.46–1.88]	1.26	[1.10–1.44]
	Immigration status						
	Native	3343	16.0 (14.8–17.3)	1.00			
	First‐ or second‐generation immigrant	1046	21.6 (19.2–24.2)	1.35	[1.14–1.60]		
	Sexual orientation						
	Heteronormativity	3980	15.6 (14.5–16.7)	1.00		1.00	
	No heteronormativity	578	33.4 (29.7–37.3)	2.14	[1.85–2.48]	1.45	[1.22–1.72]
	Academic performance						
	Good or average	3767	15.0 (13.9–16.2)	1.00		1.00	
	Poor	556	31.8 (28.1–35.8)	2.12	[1.84–2.45]	1.51	[1.34–1.71]
Psychosocial factors	Quality of family relationships						
	Very good or good	4044	14.2 (13.2–15.3)	1.00		1.00	
	Fair, poor, or very poor	514	46.5 (42.2–50.8)	3.28	[2.90–3.70]	1.88	[1.66–2.10]
	Bullying						
	No	3901	15.5 (14.4–16.7)	1.00		1.00	
	Yes	657	31.8 (28.4–35.5)	2.06	[1.78–2.37]	1.45	[1.24–1.80]
	Sexual violence						
	No	3969	15.9 (14.8–17.1)	1.00		1.00	
	Yes/prefer not to answer	541	30.5 (26.8–34.5)	1.91	[1.67–2.19]	1.21	[1.04–1.41]
Health behaviors	Physical activity						
	Meets WHO recommendations	2615	14.0 (12.7–15.4)	1.00		1.00	
	Does not meet WHO recommendations	1943	23.0 (21.2–24.9)	1.64	[1.47–1.84]	1.23	[1.09–1.40]
	Sleep quality						
	Very good or good	3337	11.9 (10.8–13.0)	1.00		1.00	
	Poor or very poor	1221	34.2 (31.5–36.9)	2.88	[2.61–3.18]	2.08	[1.85–2.35]
	Diet quality						
	Healthy or needs changes	4287	17.1 (16.0–18.2)	1.00		1.00	
	Unhealthy	271	29.9 (24.7–35.6)	1.75	[1.45–2.11]	1.24	[1.01–1.51]
	Hazardous alcohol use						
	No	3965	17.7 (16.5–18.9)	1.00			
	Yes	593	18.9 (15.9–22.2)	1.07	[0.93–1.22]		
	Hazardous cannabis use						
	No	4426	17.5 (16.4–18.6)	1.00			
	Yes	132	29.5 (22.4–37.9)	1.70	[1.24–2.31]		
	Problematic internet use						
	No	3730	15.0 (13.9–16.1)	1.00		1.00	
	Yes	809	30.4 (27.3–33.7)	2.03	[1.77–2.34]	1.28	[1.11–1.47]

**Table 3:** Prevalence of poor mental well‐being and crude and adjusted prevalence ratios (PR and aPR) for each factor associated with poor mental well‐being in school‐aged girls in Central Catalonia in the 2021–2022 academic year (*N* = 4707)

		*N*	% and 95% CI	PR	95% CI	aPR	95% CI
Overall health	Self‐perceived overall health						
	Excellent or very good	4007	35.2 (33.7–36.7)	1.00		1.00	
	Regular, poor, or very poor	700	78.0 (74.8–80.9)	2.22	[2.07–2.37]	1.43	[1.32–1.55]
Contextual factor	Size of municipality						
	Rural	2425	41.8 (39.8–43.7)	1.00			
	Urban	2172	41.2 (39.2–43.3)	0.99	[0.90–1.08]		
Axes of inequality	Grade level						
	Second and fourth course of CSE	3201	41.1 (39.4–42.9)	1.00			
	Second course of PCSE and ILTC	1506	42.4 (40.0–44.9)	1.03	[0.94–1.13]		
	Subjective SEP						
	Advantaged or Average	3050	36.2 (34.5–37.9)	1.00		1.00	
	Deprived	1657	51.5 (49.1–53.9)	1.42	[1.31–1.54]	1.09	[1.02–1.17]
	Immigration status						
	Native	3410	38.2 (36.6–39.9)	1.00		1.00	
	First‐ or second‐generation immigrant	1104	49.6 (46.7–52.6)	1.30	[1.21–1.40]	1.12	[1.04–1.21]
	Sexual orientation						
	Heteronormativity	3154	36.3 (34.6–38.0)	1.00		1.00	
	No heteronormativity	1553	52.2 (49.7–54.7)	1.44	[1.32–1.57]	1.15	[1.07–1.25]
	Academic performance						
	Good or average	4115	38.3 (36.8–39.8)	1.00		1.00	
	Poor	383	66.8 (62.0–71.4)	1.74	[1.62–1.88]	1.19	[1.10–1.30]
Psychosocial factors	Quality of family relationships						
	Very good or good	3611	32.9 (31.4–34.5)	1.00		1.00	
	Fair, poor, or very poor	1096	70.0 (67.2–72.6)	2.13	[2.00–2.26]	1.57	[1.47–1.66]
	Bullying						
	No	3804	37.7 (36.2–39.3)	1.00		1.00	
	Yes	903	57.7 (54.4–60.9)	1.53	[1.43–1.63]	1.22	[1.15–1.30]
	Sexual violence						
	No	2033	33.1 (31.0–35.1)	1.00		1.00	
	Yes/prefer not to answer	2623	48.0 (46.1–50.0)	1.45	[1.36–1.56]	1.12	[1.04–1.20]
Health behaviors	Physical activity						
	Meets WHO recommendations	1752	34.8 (32.6–37.1)	1.00		1.00	
	Does not meet WHO recommendations	2955	45.5 (43.8–47.4)	1.31	[1.21–1.42]	1.15	[1.06–1.24]
	Sleep quality						
	Very good or good	2775	29.4 (27.7–31.1)	1.00		1.00	
	Poor or very poor	1932	59.0 (56.8–61.2)	2.01	[1.87–2.15]	1.52	[1.41–1.64]
	Diet quality						
	Healthy or needs changes	4450	40.5 (39.0–41.9)	1.00		1.00	
	Unhealthy	257	60.3 (54.2–66.1)	1.49	[1.35–1.65]	1.15	[1.03–1.28]
	Hazardous alcohol use						
	No	3816	40.3 (38.8–41.9)	1.00			
	Yes	891	46.9 (43.7–50.2)	1.16	[1.08–1.26]		
	Hazardous cannabis use						
	No	4576	41.1 (39.7–42.6)	1.00			
	Yes	131	55.7 (47.1–64.0)	1.35	[1.15–1.60]		
	Problematic internet use						
	No	3368	35.9 (34.3–37.5)	1.00		1.00	
	Yes	1331	55.9 (53.2–58.5)	1.56	[1.46–1.66]	1.19	[1.11–1.27]

Finally, Table 4 shows the cumulative effect score of risk exposure to overall health, axes of inequality, psychosocial factors and health behaviors associated with poor mental well‐being across genders. The cumulative effect score revealed a cumulative effect of the presence of multiple risk factors of mental health and its significant impact on poor mental well‐being. The data clearly show a gradient where students show stronger associations with poor mental well‐being as they are exposed to a higher number of risk factors. This pattern is consistent across genders. Boys exposed to 10 health and social factors combined had 24.52 times a statistically significant higher prevalence of poor mental well‐being ([95% CI 15.75–38.17], *p* < .001) than those exposed to none. As for girls, those exposed to 11 health and social factors combined had 16.23 times a statistically significant higher prevalence of poor mental well‐being ([95% CI 10.02–26.28], *p* < .001) compared to those exposed to none.

**Table 4:** Cumulative score of health and social factors associated with poor mental well‐being across gender

No. of health and social factors^a^	Boys			Girls		
	*N*	PR (95% CI)	*p*‐value	*N*	PR (95% CI)	*p*‐value
1	1051	1.59 (1.02–2.46)	.**040**	514	2.56 (1.51–4.32)	**<.001**
2	976	2.96 (1.81–4.86)	**<.001**	801	4.07 (2.44–6.79)	**<.001**
3	736	3.96 (2.43–6.46)	**<.001**	825	5.10 (3.10–8.38)	**<.001**
4	457	8.75 (5.65–13.54)	**<.001**	745	7.56 (4.66–12.26)	**<.001**
5	244	10.75 (6.82–16.94)	**<.001**	527	9.21 (5.64–15.03)	**<.001**
6	116	13.32 (8.46–20.97)	**<.001**	282	12.03 (7.39–19.57)	**<.001**
7	49	18.52 (11.67–29.37)	**<.001**	206	12.76 (7.85–20.75)	**<.001**
8	20	18.39 (11.02–30.68)	**<.001**	102	14.16 (8.64–23.21)	**<.001**
9	6	20.43 (11.69–35.73)	**<.001**	43	15.48 (9.56–25.10)	**<.001**
10	3	24.52 (15.75–38.17)	**<.001**	18	15.33 (9.36–25.10)	**<.001**
11				4	16.23 (10.02–26.28)	**<.001**

*p*‐values highlighted in bold indicate Prevalence Ratios that are statistically significant.

^a^ 613 boys and 211 girls reported not being exposed to any of the factors.

## Discussion

The study aimed to analyze the cumulative effect of risk exposure to health and social factors associated with poor mental well‐being in school‐aged adolescents in Central Catalonia. Risk factors were grouped according to the conceptual framework for public mental health (Dykxhoorn et al., 2022), including axes of inequality, psychosocial factors (quality of family relationships, bullying, and sexual violence), and health behaviors.

Our findings showed that 17.8% of boys and 41.6% of girls aged 12–18 years old reported having poor mental well‐being. We also found that mental well‐being was multifaceted, associated with multiple axes of inequality (Muntaner, Eaton, Diala, Kessler, & Sorlie, 1998), overall health, psychosocial factors, and health behaviors. Moreover, we found the existence of a dose–response gradient indicating that the prevalence of poor mental well‐being rises with an increasing accumulation of health and social factors.

Our study found a higher prevalence of poor mental well‐being (29.9%) compared to the 2021 Spanish Barometer of Youth, Health, and Well‐being, where 17.7% of adolescents aged 15–19 reported frequently experiencing psychological, psychiatric or mental health problems in the last 12 months (Sanmartín et al., 2022). This difference can be contextualized, given our study assessed mental well‐being rather than mental ill‐health. These two measures are distinct but overlapping constructs, with different levels of stability and predictors across childhood and adolescence (Patalay & Fitzsimons, 2018). Thus, it is predictable to find differences from broader national estimates of mental health difficulties. Despite this, gender differences in the prevalence of poor mental well‐being were consistent with Spanish estimations and vast international evidence (Campbell, Bann, & Patalay, 2021; Sanmartín et al., 2022), and with previous studies assessing low mood (Carrilero, Dalmau‐Bueno, & García‐Altés, 2021; González‐Casals et al., 2023; Vázquez Fernández et al., 2013).

In agreement with previous extensive research on the study of the determinants of mental health and the conceptual framework for public mental health (Dykxhoorn et al., 2022), we observed multiple health determinants associated with poor mental well‐being across both genders, among which: axes of inequality such as having a low socioeconomic position (SEP) (Carrilero et al., 2021; Vukojević et al., 2017), having nonheteronormative sexual orientation (Meyer, 2003), and reporting low academic performance (Agnafors, Barmark, & Sydsjö, 2021); psychosocial factors such as having suffered bullying or sexual abuse (Analitis et al., 2009; Bentivegna & Patalay, 2022; Rigby, 2003), and low‐quality family relationships (Silva et al., 2021); reporting low self‐perceived health (Codinach‐Danés et al., 2024); some health behaviors such as low‐quality sleep, following an unhealthy diet, not meeting the WHO recommendations for physical activity (Hutchesson et al., 2023; Wickham et al., 2020), and problematic internet use (Lam & Peng, 2010; Vázquez Fernández et al., 2013). In contrast to other studies, no statistically significant associations were found with the size of the municipality (Dursun et al., 2020; Jiang, Sun, & Marsiglia, 2016), and inconsistent results were found regarding problematic drug use (Codinach‐Danés et al., 2024; Davoren, Shiely, Byrne, & Perry, 2015; Mangot‐Sala et al., 2019).

Our findings show a gradient, indicating that adolescents are increasingly susceptible to poor mental well‐being as they are associated with a greater number of health and social factors. The existence of such gradient underscores the cumulative effect of health determinants on adolescent mental health, challenging conventional approaches that tackle these determinants in isolation. These findings are consistent with studies exploring cumulative risk exposure within many mental health problems such as anxiety, depression, suicidal ideation, trauma symptoms, and externalizing symptoms (Appleyard et al., 2005; Deater‐Deckard et al., 1998; Demkowicz et al., 2021; Raviv et al., 2010; Wang et al., 2024). For this reason, while the study may initially appear less groundbreaking, it is among the first to incorporate a large, representative sample of adolescents from both rural and urban settings, thereby providing a more comprehensive and accurate quantification of the cumulative effects of multiple risk factors on mental well‐being. This approach is of considerable significance for clinical practice, as it highlights the cumulative effect of various risk factors, aligning with research that shows how compounded risk factors can amplify psychological distress and mental health issues (Appleyard et al., 2005; Evans, Li, & Whipple, 2013). Thus, in addition to treating illness, clinicians should also consider the broader context in which that illness developed. By prioritizing modifiable health and social risk factors, clinicians can adopt more holistic intervention methods by addressing risks before they become embedded as more severe issues, which can be more impactful in creating positive mental health outcomes (Halfon & Hochstein, 2002). Addressing even a single modifiable factor has been shown to reduce distress, fostering a “positive feedback loop” that enables further action on additional risk factors, in line with findings that sequential and integrative approaches often yield better long‐term adaptive behavior outcomes (Masten & Cicchetti, 2010). Consequently, this method empowers clinicians and preventive specialists to look beyond immediate causes of distress – which are often the most complex to modify – and instead focus on a progressive approach that builds upon achievable changes.

Beyond clinical interventions, adolescence is a critical developmental stage in which social and environmental factors strongly influence mental health outcomes, and high schools constitute a central social environment in which young people are embedded (Hinze et al., 2024). The cumulative burden of risk factors observed in our study reinforces the importance of school‐based approaches aimed at promoting adolescent mental well‐being. One framework that has gained increasing relevance is Health Promoting Schools, which integrates the socio‐health dimension of the educational center with the creation of salutogenic environments (Turunen, Sormunen, Jourdan, Von Seelen, & Buijs, 2017). These include accessibility to families, inclusivity, support shaping socialization processes, capacity for early detection and intervention, and a role as community hubs (Grupo de trabajo de Salud Escolar y estilos de vida de las CC. AA, & Ministerio de Educación, Formación Profesional y Deportes, 2023; Lee, Lo, Li, Keung, & Kwong, 2020). Through these mechanisms, schools emerge as crucial settings that can contribute not only to academic development but also psychosocial well‐being, thereby fostering resilience and reducing the risk of mental health problems through the mitigation of health‐related and social factors more amenable to change through public health actions (Pulimeno, Piscitelli, Colazzo, Colao, & Miani, 2020). School‐based programs have the potential to improve both health and education outcomes through sustained collaboration between education and health sectors to address the complex social determinants that shape adolescent development, and especially those coming from a disadvantaged background (Kolbe, 2019). Schools for Health in Europe, too, has outlined guidelines for creating successful mental health promotion initiatives in schools. They advocate for a holistic and contextual approach that promotes health and well‐being by making organizational changes to strengthen physical and social environments (O'Toole & Darlington, 2021).

Finally, regarding gender inequalities in mental well‐being, girls consistently showed a higher prevalence of poor mental well‐being across all factors, indicating a higher overall burden. However, boys showed greater prevalence ratios, meaning that the relative increase in poor mental well‐being associated with specific risk factors was higher for them. This apparent contradiction may be due to differences in baseline risk: since girls already reported a higher baseline prevalence of poor mental well‐being and were exposed to more associated factors, the relative impact of additional associated factors may appear smaller when compared to boys, who start from a lower baseline. This lower baseline may make boys more vulnerable to the incremental effects of certain exposures, even if their absolute levels of well‐being remain lower. This may reflect broader gendered patterns of inequality which determine access to resources and opportunities for good health (Comisión Para Reducir Las Desigualdades Sociales En Salud En España, 2012), resulting in girls being exposed to a greater number of health‐related risk factors. It is a fact that boys and girls differ in the experiences and stressors they face as they transition from early adolescence and find themselves in patriarchal cultural processes of gender socialization that influence development, opportunities, and health, which might not have been captured in this study. For instance, gender‐based discrimination is a reality for most girls, and elevated levels of gender discrimination are associated with greater internalizing symptoms (e.g., anxiety, depressive symptoms, and loneliness) (Rogers, Cook, & Guerrero, 2022). This is one example of the structural pressures of gender inequality that weigh on girls. As they already carry a heavier burden of determinants that influence poor mental health, this could explain why additional risks may not increase the prevalence ratio as much as they would in a population with a lower baseline risk, such as in boys. Other authors have found boys' and girls' coping mechanisms tend to differ: girls reported higher proportions of seeking social support and problem‐solving strategies, while boys reported a more avoidant coping style (Eschenbeck, Kohlmann, & Lohaus, 2007). The use of more adaptive strategies to cope with adversity could also explain the lower prevalence ratios in girls. Another study, however, found that girls' coping competence was considerably lower compared to boys (Haugan, Frostad, & Mjaavatn, 2021). For this reason, it is crucial to examine how coping mechanisms can influence the recognition, expression, and handling of emotional distress.

### Strengths and limitations

The main strength of this study is the availability of a large sample of school‐aged adolescents in various territories from Central Catalonia, including both urban and rural populations. Although the representation is limited to Central Catalonia and does not include all adolescents, particularly those outside formal education, it nevertheless captures multiple profiles and facilitates the inclusion of adolescents living in rural areas, as most research is typically conducted within urban populations. A key strength of the study lies in its large, population‐based design, encompassing nearly 70% of adolescents enrolled in the target academic levels throughout Central Catalonia. This ensures a high degree of representativeness and supports the generalizability of the results while reducing sampling bias.

However, our study also has some limitations. First, the cross‐sectional design of the study only allowed us to establish associations between the exposure variables and poor mental well‐being, rather than cause–effect relationships. These associations are useful for assessing prevalence and gathering preliminary evidence for future advanced studies (Wang & Cheng, 2020). However, given the lack of systematic control over experimental error, the findings should be interpreted with caution and regarded as indications of areas worthy of further research. Second, the data were collected using a self‐reported survey, which allowed for social desirability bias; therefore, the data may be inaccurate. Nevertheless, self‐reported surveys are commonly used in this type of study due to their ease of administration and low cost. Moreover, the survey was created based on similar surveys with high validity and reliability. Third, the selection of variables was constrained by the items available in the questionnaire. While the significance of other pertinent factors, including discrimination, harsh parenting, and experiences of child abuse and neglect, is acknowledged, these were not evaluated due to ethical and practical limitations concerning the assessment of highly sensitive experiences in school‐based surveys. However, related indicators such as bullying victimization and lifetime exposure to sexual violence were included. It is imperative to consider these limitations when interpreting the study's scope, as there is a possibility that certain key determinants of adolescent mental health may not have been fully captured. Fourth, given the exploratory nature of the bivariate analyses, it was deemed unnecessary to apply any correction for multiple testing. Consequently, findings derived from these unadjusted models should be interpreted with caution. The primary conclusions of the study are derived from multivariable models, which are informed by both theoretical frameworks and empirical evidence, thereby providing more robust and adjusted estimates. Finally, we have only been able to generate a cumulative effect score representing the number of factors that cumulatively affect poor mental well‐being, as has been done elsewhere (Salazar et al., 2022; Wu et al., 2015). Thus, a syndemic analysis based on the results of this study would be necessary to study the different interactions that could occur between the different factors, and consequently, their exacerbating effect on poor mental well‐being (Knol et al., 2011). This means looking beyond the additive models of disease and thinking about the determinants of health with a focus on their interactions (Mendenhall & Singer, 2020; Singer et al., 2020). Additionally, our statistical analysis has not allowed us to indicate which factors were behind the cumulative score. Nevertheless, we have been able to demonstrate the dose–response effect of factors that were associated with poor mental well‐being independently.

## Conclusions

This study adds to the increasing knowledge of adolescent mental health by showing that the coexistence of multiple forms of inequality, overall health, psychosocial factors, and health behaviors has a cumulative impact on adolescents' mental well‐being.

Our study is the first to investigate mental health in a large sample of adolescents from both rural and urban areas, incorporating a variety of health determinants within a conceptual framework specifically designed to explain adolescent mental health. This research has enabled us to quantify the cumulative effect of different factors and identify which variables are most strongly associated with mental health outcomes. In clinical practice, this adds evidence to the importance of focusing not only on the mental health symptoms themselves, but also on the modifiable health and social factors which can trigger positive improvements in adolescents' overall mental health. This approach will enhance our ability to visualize and understand social inequalities in adolescent mental health, also aiding in the development of intersectional interventions that promote mental well‐being in adolescents.

## Conflict of interest statement

The authors have declared that they have no competing or potential conflicts of interest.

## Funding information

This project has been funded by the Plan Nacional Sobre Drogas (2021|86) and the Subprograma de Desigualdades sociales en la Salud y en los comportamientos de Salud de las personas jóvenes de España del Centro de Investigación Biomédica en Red de Epidemiologia y Salud Pública (CIBERESP) (ESPCP41/2022).

## Ethical considerations

The project was approved by the Research Ethics Committee of the Universitat de Vic‐Universitat Central de Catalunya (UVic‐UCC) (approval reference number: 96/2019), on September 24, 2019. Informed consent was appropriately obtained for all participants aged 14 or over, whereas parental consent was obtained from participants' legal guardians for those under 14 years old.

## Supporting information


**Appendix S1.** STROBE Statement – Checklist of items that should be included in reports of cross‐sectional studies.

## Data Availability

The data that support the findings of this study are available from the corresponding author upon reasonable request.
